# Progress in treatment of neuromyelitis optica spectrum disorders (NMOSD): Novel insights into therapeutic possibilities in NMOSD

**DOI:** 10.1111/cns.13836

**Published:** 2022-04-15

**Authors:** Mingchao Shi, Fengna Chu, Tao Jin, Jie Zhu

**Affiliations:** ^1^ Neuroscience Center Department of Neurology The First Hospital of Jilin University Changchun China; ^2^ Department of Neurobiology, Care Sciences & Society Division of Neurogeriatrcs Karolinska Institutet Karolinska University Hospital Solna Stockholm Sweden

**Keywords:** central nervous system, demyelinating, inflammation, neuromyelitis optica spectrum disorders, treatment

## Abstract

Neuromyelitis optica spectrum disorder (NMOSD) is a rare autoimmune inflammatory demyelinating disorder of the central nervous system (CNS), which is a severely disabling disorder leading to devastating sequelae or even death. Repeated acute attacks and the presence of aquaporin‐4 immunoglobulin G (AQP4‐IgG) antibody are the typical characteristics of NMOSD. Recently, the phase III trials of the newly developed biologicals therapies have shown their effectiveness and good tolerance to a certain extent when compared with the traditional therapy with the first‐ and second‐line drugs. However, there is still a lack of large sample, double‐blind, randomized, clinical studies to confirm their efficacy, safety, and tolerability. Especially, these drugs have no clear effect on NMOSD patients without AQP4‐IgG and refractory patients. Therefore, it is of strong demand to further conduct large sample, double‐blind, randomized, clinical trials, and novel therapeutic possibilities in NMOSD are discussed briefly here.

## Graphical abstract

Several drugs with different mechanisms and targets have been applied in clinic for therapy of NMOSD patients, included first‐line, second‐line, and new biologicals therapies. Other novel insights into therapeutic possibilities in NMOSD include targeting Th17 cells and blocking IL23/IL17/Th17 pathway and stem cell therapies, etc.

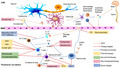

## 1 INTRODUCTION

Neuromyelitis optica spectrum disorder (NMOSD) is an autoimmune inflammatory demyelinating disorder of the central nervous system (CNS). NMOSD mainly affects the optic nerve and spinal cord^1^, ^2^, ^3^ and its lesions also involve the brain stem and cerebrum in most cases by repeated brain attacks, manifesting as optic neuritis, myelitis, and certain brain and brainstem syndromes.^4^ NMOSD is a severely disabling disorder leading to devastating sequelae, such as permanent blindness or paralysis or even death.^2^, ^5^ Simultaneously, NMOSD patients are complicated with severe persistent neuropathic pain, and about half of the patients had severe pain, which seriously affected the quality of life of patients.^6^, ^7^

The epidemiological investigations show that NMOSD is considered as a rare disorder and worldwide prevalence was low with 0.3–0.5–4.4/100,000 population^8^, ^9^ estimating global pooled prevalence with 1.82 per 100,000 people.^10^ Blacks have the highest prevalence, followed by Asians, and whites have the lowest, suggesting varying prevalence seen in different racial populations.^4^, ^11^ In the general population, the incidence of NMOSD ranged from 0.053 to 0.4 per 100,000 per year and both the incidence and prevalence of NMOSD were different worldwide.^10^

Previously, it was difficult to identify and diagnose NMOSD due to its intricate clinical and neuroimaging manifestations, which were indistinguishable from multiple sclerosis (MS). Excitingly, the autoantibodies that target the aquaporin‐4 (AQP4) water channel protein were discovered and evidenced to have pathogenic potential for neuromyelitis optica (NMO) in 2004,^12^, ^13^ providing a better way to identify and diagnose NMOSD based on the presence of AQP4 immunoglobulin G (AQP4‐IgG).^2^, ^14^ There is growing evidence that the pathogenic factor, AQP4‐IgG antibody in serum and cerebrospinal fluid (CSF), has been observed in about 70% of NMOSD patients only.^15^, ^16^, ^17^ However, 20–40% clinically diagnosed NMOSD patients still lack detectable AQP4‐IgG.^18^, ^19^ Thus, another autoantibody of NMOSD, myelin oligodendrocyte glycoprotein (MOG)‐IgG, has also been proposed as a candidate biomarker for NMOSD in AQP4‐IgG‐seronegative patients, named as MOG‐IgG‐associated disease (MOGAD).^20^ Moreover, both MOGAD and NMOSD are responsible for clinically distinct subsets of optic neuritis (ON).^21^, ^22^ Usually, when compared NMOSD AQP4‐IgG seropositive with AQP4‐IgG seronegative/MOG‐IgG positive, it exhibited a different pathological mechanism, but the latter showed a similar clinic and a better prognosis.^18^ Based on NMOSD's new diagnostic criteria, NMOSD is stratified by serostatus into NMOSD with AQP4‐IgG and without AQP4‐IgG, and combined with the necessary requirements and the core clinical characteristics.^21^, ^22^ It is also required to detect MOG‐IgG in NMOSD patients without AQP4‐IgG to determine or exclude MOGAD. However, it is still difficult to correctly diagnose NMOSD without AQP4‐IgG/MOG‐IgG relying on the clinical and imaging manifestations so far.^23^ This brings challenges to the diagnosis of NMOSD and need to be carefully differentiated from MS, MOGAD, and other demyelinating diseases in the CNS.^2^

Majority of AQP4‐IgG is produced in peripheral lymphoid tissues, so the levels of AQP4‐IgG in serum are much higher than in CSF. AQP4‐IgG contributes to the pathogenesis of NMOSD through activating complement and promoting disruption of astrocytic membranes and blood–brain barrier (BBB) causing dysfunction of brain water movement, resulting in a series of pathophysiological changes.^24^ However, not all NMOSD patients have the pathogenic AQP4‐IgG,^25^, ^26^ which raises a question whether AQP4‐IgG is the unique pathogenic factor for NMOSD. Therefore, the pathogenesis of NMOSD may be multifactorial and is more complex than a simple pathogenic AQP4‐IgG would suggest, which remains unclear.

So far, all efforts made in the treatments of NMOSD with targeting the pathogenic factor, AQP4‐IgG, and its related molecules have obtained a remarkable progress and success in therapy of some cases. Unfortunately, these therapies for NMOSD are limited and only partially effective in most cases. Thus, it is crucial to study and explore the effective treatment methods and medicines in NMOSD. In the present review, we summarized briefly the therapeutic outcomes in NMOSD patients during the last decade and analyzed the effects and adverse events of these drugs in different races and populations. Since current advent of novel treatments provided promising medications for the treatment of NMOSD, we discussed the potential therapeutic effects with novel medications in NMOSD and further research is needed to develop treatment guidelines.

## 2 CURRENT THERAPIES IN NMOSD

At present, several drugs with different mechanisms and targets have been applied in clinic for therapy of NMOSD patients, which have been recommended as the first‐line therapy with glucocorticoids, azathioprine (AZA), and rituximab (RTX), as well as the second‐line therapy with other immunosuppressive drugs, such as methotrexate, mycophenolate mofetil (MMF), and mitoxantrone in NMOSD.^27^, ^28^ However, MMF is also recommended as the first‐line therapy in NMOSD by some groups.^28^, ^29^

**FIGURE 1:**
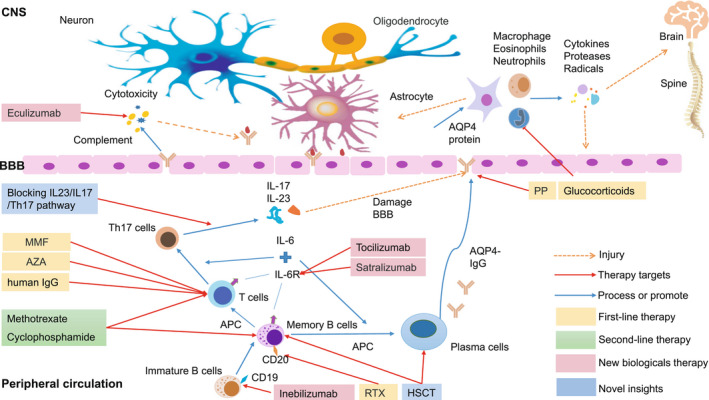
New biological therapies in NMOSD. Interleukin‐6 (IL‐6) as a T‐cell‐derived cytokine induced B lymphocyte differentiation into immunoglobulin (IgG)‐producing plasma cells, which also regulates the differentiation of native CD4+ T cells into pathogen‐specific effector T‐helper cell 17 (Th17) that release both IL‐17 and IL‐23 and increase the permeability of blood–brain barrier (BBB).^106^, ^107^ Then, aquaporin‐4 (AQP4)‐IgG produced from the peripheral lymphoid tissues passes BBB and enters the central nervous system (CNS) binding to the AQP4 proteins expressed on astrocytes, afterwards the complex can active the complement damaging and promoting disruption of astrocytic membranes and BBB, causing dysfunction of brain water movement, as well as recruiting inflammatory cells like macrophages, neutrophils, and eosinophils entering into the CNS. The inflammatory cells could release proteases, cytokines, and radicals that further injure CNS parenchymal, optic nerve, and blood vessels. Astrocyte damage leads to loss of support for oligodendrocytes and neurons, which ultimately leads to loss of myelin sheath. Several drugs with different mechanisms and targets have been applied in clinic for therapy of NMOSD patients, which have been recommended as the first‐line therapy with glucocorticoids and immunosuppressants, such as azathioprine (AZA), mycophenolate mofetil (MMF), and rituximab (RTX). Usually, when glucocorticoids pulse therapy failed in NMOSD, plasmapheresis (PP) and human IgG were chosen as the second option for therapy of the acute NMOSD. The second‐line therapy includes other immunosuppressive drugs, such as methotrexate and cyclophosphamide. Now the major progress in treatment of NMOSD is developing the exciting new biologicals, such as anti‐AQP4‐IgG agents (inebilizumab as a targeting CD19 antibody), anti‐IL‐6 receptor antibodies (tocilizumab and satralizumab), and complement inhibitor (eculizumab). Other novel insights into therapeutic possibilities in NMOSD include targeting Th17 cells and blocking IL23/IL17/Th17 pathway and stem cell therapies, etc. Abbreviations: APC: Antigen‐presenting cells; AQP4: Aquaporin‐4; AZA: Azathioprine; BBB: Blood–brain barrier; CNS: Central Nervous System; HSCT: Hematopoietic stem cell transplantation; IgG: Immunoglobulin G; IL‐6: Interleukin‐6; IL‐6R: Interleukin‐6 receptor; MMF: Mycophenolate mofetil; NMOSD: Neuromyelitis optica spectrum disorders; PP: Plasmapheresis; RTX: Rituximab; Th17: T‐helper cell 17

Now the major progress in treatment of NMOSD is developing the exciting new biological therapies, such as inebilizumab and ocrelizumab targeting B cells, anti‐interleukin‐6 (IL‐6) receptor antibodies (tocilizumab and satralizumab), and complement inhibitor (eculizumab), which bring hope and dawn to treat NMOSD.^5^, ^27^, ^28^, ^30^, ^31^, ^32^, ^33^ To date, nevertheless, there is no approved therapy or curative treatments in NMOSD.^5^, ^27^ Standard or traditional treatments with the first‐ and second‐line therapies in most patients with NMOSD could prevent acute attacks and maintain remission. However, the standard treatments were ineffective in both patients with AQP4‐IgG seronegative and the refractory patients. Currently, the consensus is that the goals of treatment of NMOSD are as follows: (1) relieving relapse‐related symptoms; (2) preventing disease relapse and keeping stable condition; and (3) symptomatic treatment. In recent years, our research work has been focusing on NMOSD,^34^, ^35^, ^36^, ^37^, ^38^ and our therapeutic view is alleviating the acute symptoms and preventing the relapses of NMOSD, the two key objectives of the therapeutic approach for NMOSD. Additionally, the role of follow‐up monitoring of AQP4‐IgG in the disease should be conducted. The therapeutic strategy in NMOSD is presented in Figure 1.

### 2.1 The first‐line therapy in NMOSD

The drugs of the first‐line therapy in NMOSD include glucocorticoids, plasmapheresis, and immunosuppressants, such as RTX, MMF, and AZA, which have been evidenced effectively in treatment of some NMOSD patients with AQP4‐IgG seropositive, manifesting in improved clinical symptoms and lowering the risk of relapse at varying degrees.

#### 2.1.1 Glucocorticoids

Glucocorticoids as the first‐line and basic therapy in NMOSD has been widely applied in clinic for treatment of NMOSD for a long time. Glucocorticoids pulse therapy was frequently used at the acute phase of NMOSD, subsequently glucocorticoids as a basic therapy combated with other immunosuppressants applied in the phases of remission and relapse of NMOSD. A study of Taiwan showed that 96 NMOSD patients treated with glucocorticoids combated with immunosuppressants at the acute phase for long‐term therapy were followed up for more than 2 years, displaying poor visual prognosis.^39^ The effect of glucocorticoids alone on NMOSD is beyond proof because most of the clinical trials in NMOSD used glucocorticoids combated with immunosuppressants.

#### 2.1.2 Plasmapheresis

So far the efficacy of plasmapheresis (PP) in NMOSD has not been proofed by large sample, randomized, controlled trials,^40^ despite reports of successful therapeutic cases in MNOSD.^41^ Usually, when glucocorticoids pulse therapy failed in NMOSD, PP and human immunoglobulin (IgG) were chosen as the second option for therapy of the acute NMOSD. It has been reported that in 15 NMOSD patients treated by glucocorticoids pulse therapy and PP add‐on, the visual acuity of the patients was remarkably improved, which is a clinical feature of NMOSD with repeated vision impairment.^38^ Compared with monotherapy with glucocorticoids, PP add‐on treatment was obviously effective in restoring vision and improving the prognosis after NMOSD relapses.^38^, ^42^, ^43^

#### 2.1.3 Immunosuppressants of the first‐line therapy in NMOSD

The efficacy of these immunosuppressants as the first‐line therapy in NMOSD during 3 years after NMOSD onset was observed in a recent study, in which 136 NMOSD patients were treated by RTX, MMF, and AZA, respectively. Treatment with RTX was obviously effective compared with MMF in improving clinical symptoms and reducing disease activity and the risk of relapse, which was irrelevant to the antibody levels. There was no difference between treatment with RTX and AZA.^28^

RTX has long been considered a first‐line treatment in NMOSD through its effect with depleting CD20 B cells.^27^ D20 molecule as a cell surface antigen expresses on pre‐B cells, mature and memory B cells, but not on the earliest B‐cell precursors or on plasma cells.^44^ RTX has been recommended for clinic application in multiple clinic observations^45^ via depleting CD20 B cells to decline the pathogenic antibody production, thereby alleviating the symptoms of disease. A study with 73 Italian NMOSD patients treated by RTX showed that RTX declined relapse rate and neurologic disability, however, did not either affect the frequencies of autoreactive B cells or reset defective early B‐cell tolerance checkpoints.^46^ It is noteworthy that treatment with RTX in NMOSD patients with AQP4‐IgG seropositive can prevent disease relapses effectively during more than 1.5 years, indicating that RTX can be recommended to maintain therapy in NMOSD patients with AQP4 antibody.^47^

Another clinical trial in 23 NMOSD patients treated with RTX was conducted for more than 40 months and AQP4‐IgG positive was found in 78% of the patients. RTX therapy significantly reduced the number of relapses in 91% cases, and annualized relapsed rate (ARR). The mean expanded disability status scale score (EDSS) also declined from 4.8 to 3.9. The results showed that RTX was well tolerated, and infections were main adverse events in 65.2% of cases, which indicated RTX effectiveness and safety in patients with NMOSD.^48^

A clinical observation in Korea reported that before RTX treatment, 9 of 12 NMOSD pregnant patients had pregnancy‐related attack, however, after initiation of RTX therapy, only in 5 of 21 pregnancies attack occurred, which implied that RTX could decline and prevent pregnancy‐related attack before pregnancy.^49^ A total of 100 Korean patients received repeated RTX treatment during a median of 67 months, afterwards follow‐up for more than 5 and 7 years, respectively. The results displayed an increasing number of patients and duration of exposure and maintained good efficacy and safety of RTX.^50^ However, most studies regarding the therapeutic effects of RTX on NMOSD were placebo controlled. Therefore, it is necessary to conduct a large sample of other therapeutic agents control studies.

Similarly, AZA and MMF decreased the risk of relapses and disability progression in 206 NMOSD patients with AQP4‐IgG seropositive in another clinical investigation.^51^ Importantly, the treatments with these immunosuppressants in NMOSD should last for 5 years at least.^52^

A recent prospective cohort study enrolling 281 Chinese NMOSD patients has revealed that both AZA and MMF evidently declined the risk of disability and relapse, resulting in NMOSD progress delay through assessing the effects of the first‐line immunotherapies over a long period of time.^53^ Another Chinese research group also reported that AZA, MMF, and lower dosages of RTX (100 mg RTX intravenous injection, once per week for 4 consecutive weeks) remarkably relieved the clinical symptoms in NMOSD patients with AQP4‐IgG seropositive and obviously lowed ARR.^54^ At the same time, their study investigated the impact of these drugs on AQP4‐IgG and side effects. The results showed MMF and lower dosages of RTX declined AQP4‐IgG titers and side effects significantly compared to AZA.^54^ Additionally, clinical trial demonstrated that RTX at lower dosage reduced the counts of CD19 B cells more effective than other dosages.^54^

In a clinic trail with the first‐line therapy in NMOSD, 198 NMOSD patients receiving treatment with AZA, MMF, or cyclophosphamide (CTX) (*n* = 119, 38, and 41, respectively) were cotreated with oral prednisone. The results showed that (1). The good first‐line therapeutic option was MMF; and (2). If AZA´s side effects were tolerable, AZA was also the first‐line drugs worth applying.^55^

However, there were some defects and shortcomings in these studies, since some drugs, such as RTX that is dominant and given first line as relapse prevention therapy in most advanced nations, and others have not been widely used in Mainland China clinics due to several reasons, thus the studies on evaluations of the efficacy of the first‐line or the second‐line therapies in NMOSD were limited. Because RTX is too expensive, its application in middle‐ and low‐income countries has been restricted.

Previously, we also conducted the clinical trials applying the first‐line therapy in NMOSD at Neuroscience Center, Department of Neurology of The First Hospital of Jilin University, in which it enrolled about more than 100 patients with NMOSD per year. Our results showed that glucocorticoids pulse therapy was effective to improve symptoms at acute phase in most NMOSD patients, and early application of glucocorticoids combined with AZA or MMF maintaining therapy declined the relapse rate clearly. Our findings further strengthened the evidence that early treatments with MMF and AZA could obviously diminish disease relapse and disability, and benefited patients.

### 2.2 The second‐line therapy in NMOSD

#### 2.2.1 Methotrexate and cyclophosphamide

Methotrexate has been also recommended as a common drug in NMOSD. A small sample size study reported that methotrexate treatment was started as an initial long‐term immunosuppressant in NMOSD patients. After 18 months of treatment, ARR in methotrexate‐treated group was significantly decreased, suggesting methotrexate being safe and effective as a single long‐term immunosuppressant in NMOSD.^56^ The similar findings have been also observed in NMOSD patients treated with methotrexate in other studies.^57^ As a few groups suggested, if NMOSD patients were failed by other treatments, methotrexate may be recommended as a therapeutic option in NMOSD patients due to its stabilization of disability and good tolerance,^55^, ^56^, ^57^ which needs further study to confirm because the studies came from a small clinical sample size.

Intravenous cyclophosphamide therapy in NMOSD was effective in both acute and chronic phases of NMOSD and cyclophosphamide combined with intravenous methylprednisolone or with PP was valuable therapeutic modality.^58^ However, when NMOSD patients treated with AZA and MMF showed serious side effects or could not tolerate AZA and MMF, cyclophosphamide was a good therapeutic choice.^55^

Although AZA and cyclophosphamide effectively reduced relapses in both NMOSD and NMOSD with connective tissue disease (CTD), cyclophosphamide was superior to AZA for declining relapses in patients of NMOSD with CTD through investigating 65 NMOSD patients with CTD in a retrospective study.^59^ In serial studies, totally 631 patients with NMOSD were followed up from 12 to 40 months to evaluate the efficacy and tolerance of immunosuppressants therapy. The results showed that the efficacy of RTX was superior to AZA in NMOSD, while patients were most tolerant to MMF and worst tolerance for cyclophosphamide therapy.^60^

In short, there are some defects and deficiencies in the above studies on the second‐line therapy in NMOSD. The main problem is the lack of large sample, randomized, long‐term clinical researches. Thus, the large sample, randomized, clinical studies are needed to make the correct conclusions to guide clinical treatments in NMOSD.

### 2.3 New biological therapies in NMOSD

In recent years, the studies of NMOSD have speeded up the breakthrough development of potentially new biological therapies in NMOSD. These new biologicals are promising and have emerged in the form of targeting B‐cell (inebilizumab and ocrelizumab, the anti‐CD19 and CD20 antibodies, respectively), anti‐IL‐6 receptor antibodies (tocilizumab and satralizumab), complement inhibitors (eculizumab), etc., which have been started to investigate their effectiveness and advent events in NMOSD via clinical trials in the randomized large samples. The clinical trial results evidenced that these new biological therapies successfully improved clinic symptoms, reduced the risk of disease relapse and had good tolerance in most NMOSD patients, especially in the patients with AQP4‐IgG positivity. However, in most clinical trials of new immunosuppressants are studied controlling with placebo, which makes it impossible to compare the efficacy of these new immunosuppressants with existing clinical first‐ and second‐line drugs in NMOSD.

#### 2.3.1 Targeting B cells

As mentioned above, AQP4‐IgG is a pathogenic factor and has been found in 70% NMOSD patients.^15^, ^17^ Generally, B‐cell subpopulation with CD19(int), CD27(high), and CD38(high) phenotypes produced AQP4‐IgG in NMOSD patients and obviously enhanced in the peripheral blood during relapse of disease.^61^ There's no doubt that B cells had contributed to the pathogenesis of NMOSD, which has confirmed by the growing evidence.^62^, ^63^

#### 2.3.2 Inebilizumab

Inebilizumab is an anti‐CD19, B‐cell‐depleting antibody that has been studied in a multicenter, consisting of 99 clinics or hospitals in 25 countries, which passed a double‐blind, randomized placebo‐controlled phase 2/3.^5^ In the study, there were 230 participants in total: 174 were treated with inebilizumab and 56 with placebo. The rates of general adverse events were 72% and 73% in inebilizumab group and placebo group, respectively. While the rates of serious adverse events were 5% in inebilizumab group and 9% in placebo group, the outcome provided the evidence that inebilizumab is a potential biological to treat NMOSD patients with lowing the risk of attack and disability of disease and safety when compared to placebo group.^5^, ^64^, ^65^

Ocrelizumab is a novel, anti‐CD20 B‐cell‐depleting antibody through deleting CD20 B cells to decrease AQP4‐IgG production, however, it preserves the capacity for B‐cell reconstitution and preexists humoral immunity.^66^ Ocrelizumab has been applied in clinic for treatment of NMOSD and MS; so far, the studies of ocrelizumab in NMOSD are relatively few and most studies focused on its therapeutic effect on MS.^64^ A total of 12 pregnant women with NMOSD received RTX/ocrelizumab 12 months before or during pregnancy. The results showed that 1 of 12 (8.3%) patients at least 6 months postpartum follow‐up experienced a relapse. Therefore, more researches of ocrelizumab on pregnancy outcomes and risks need to be investigated and it is also necessary to conduct the clinical study on ocrelizumab´s effects in a large sample size.^67^

#### 2.3.3 ANTI‐IL‐6 receptor antibodies

In recent years, two anti‐IL‐6 receptor antibodies, tocilizumab (TCZ) and satralizumab, with the potential therapeutical effects have been studied in active NMOSD. IL‐6 involved in the pathophysiology of NMOSD has been confirmed by the clinical and preclinical data.^31^, ^68^, ^69^, ^70^ A multicenter, randomized, phase 2 trial has been conducted at six hospitals in China and 118 patients with NMOSD were enrolled, of whom 59 were treated with TCZ and AZA, respectively. Compared to AZA, TCZ was an evidently safe and effective therapy to prevent relapses of NMOSD.^31^ In addition, a retrospective study in NMOSD patients treated with TCZ at Toulouse University Hospital was analyzed. All seven patients treated with TCZ, who were at active phase of NMOSD and suffered from the severe side effects caused by other immunosuppressant treatments, were relapse free during TCZ treatment period. Their result indicated that TCZ seemed effective in patients with refractory NMOSD.^71^

A meta‐analysis collected the relevant studies published prior to May 2020 from the PubMed, Cochrane Library, and clinicaltrials.gov databases to investigate the efficacy and safety of TCZ in NMOSD. The results showed that all 89 NMOSD patients treated with TCZ had significantly lower ARR and a close association between proportion of relapse‐free NMOSD and TCZ treatment was found. Also, the side effects in MNOSD patients treated with TCZ were mild, recommending application of TCZ therapy in NMOSD, which is safe and effective.^68^, ^69^

Blocking IL‐6 signaling with TCZ has been demonstrated as effective to treat patients with refractory NMOSD.^72^ Especially, TCZ could obviously reduce pain severity in two trials and fatigue scores in patients with NMOSD refractory to standard drugs in a trial.^73^ Lotan et al. investigated 12 NMOSD patients treated by subcutaneous TCZ at least for 6 months and found that the effectiveness of subcutaneous TCZ treatment was similar to intravenous administration in NMOSD, which was a more convenient at‐home administration.^73^ The evidence suggested that TCZ may be a promising drug to prevent acute onset in NMOSD patients.^72^

In 2020, satralizumab, as a humanized monoclonal antibody, became the third compound entering the US market to treat NMOSD by targeting IL‐6 receptor. In a phase 3 trial, NMOSD patients treated with subcutaneous or intravenous satralizumab monotherapy had a lower risk of relapse than placebo group on the primary endpoint of the relapse rate. The reason is due to satralizumab targeting IL‐6 receptors, adding to basic immunosuppressant treatment showed that relapse time was longer than placebo group. But satralizumab did not show effectiveness to relieve pain and fatigue in NMOSD.^74^ However, the occurrence of adverse events in both satralizumab and TCZ treatments was mild and comparable to AZA and placebo group, indicating satralizumab has good safety and efficacy.^70^ Additionally, the clinical trials with satralizumab were conducted in both AQP4‐IgG‐seropositive and ‐seronegative patients. Unfortunately, the results in the AQP4 IgG‐seronegative subgroup did not reveal a lower risk of relapse, suggesting that satralizumab was ineffective probably in such patients.^32^ Satralizumab is a potentially valuable treatment drug in NMOSD patients with AQP4‐IgG seropositive.^32^, ^74^

In short, therapy with TCZ and satralizumab, anti‐IL‐6 receptor antibodies in NMOSD, achieved the promising results. In order to further confirm the therapeutic effect of TCZ and satralizumab on NMOSD, it is necessary to conduct randomized, larger‐scale trials.

#### 2.3.4 Complement inhibitor (Eculizumab)

The inflammation and astrocytic injury in the CNS are mainly through complement activation after binding of AQP4‐IgG autoantibody in NMOSD, which is considered a determinant pathogenic factor.^30^, ^75^ To date, eculizumab, a terminal complement inhibitor, has been approved by US, EU, and Japan to treat AQP4 IgG‐seronegative NMOSD.

One hundred and forty‐three NMOSD patients participated in a randomized, double‐blind, time‐to‐event trial for treatment with intravenous eculizumab or placebo. AQP4 IgG‐positive NMOSD treated with eculizumab displayed an obviously lower risk of relapse than the patients treated with placebo; unfortunately, eculizumab treatment did not impact disability progression.^30^, ^76^ However, an inconsistent result was seen in a study from the same group, in which 14 NMOSD patients receiving eculizumab not only significantly showed lowed attack frequency but also improved neurological disability in active NMOSD.^75^ Thus, it is deserving and necessary to conduct further investigations in larger samples and randomized studies.^65^, ^75^

During eculizumab treatment period, the levels of serum C3 and C4 in patients did not change; nevertheless, 50% hemolytic complement (CH50) level clearly reduced without deterioration of the disease, suggesting that it is possible to monitor eculizumab efficacy via detection of serum CH50 level and CH50 may be a biomarker of eculizumab treatment.^77^

Eculizumab has been proofed to be useful in therapy of intractable NMOSD through the clinical trials described as the above. However, Paul et al. called on clinicians alerting to the risk of eculizumab‐induced infections, such as meningococcal infection during eculizumab treatment process.^78^ The same therapeutic efficacy of eculizumab was also found in another investigating study, however, an opposite finding was noted that eculizumab therapy did not appear to clearly increase serious infections.^76^

During the past 6 years, several key worldwide studies on NMOSD treatments with new biological antibodies, including inebilizumab, satralizumab, and eculizumab, have been launched based on their unique therapeutic effects and mechanisms. All of the trials were double‐masked and placebo‐controlled studies that showed a clear benefit with each approach. To date, the roles of these antibodies targeting and depleting B cells (rituximab, inebilizumab, and ocrelizumab) or blocking IL‐6 signaling (tocilizumab and satralizumab) or complement inhibitor (eculizumab) have been confirmed to have superior efficacy in diminishing NMOSD activity and inhibiting disability progression compared to placebo. In head‐to‐head studies, rituximab and tocilizumab were also superior to AZA.^79^ Simultaneously, all these antibodies have evidence to show good safety and tolerance with a lower rate of adverse events. However, the therapeutic effects of these antibodies appeared only in AQP4‐IgG‐seropositive patients, not in AQP4‐IgG‐seronegative patients, indicating therapeutic response different in two groups.^80^ Despite this, these antibodies have been demonstrated to be effective and promising therapeutic interventions in NMOSD.^81^

## 3 NOVEL INSIGHTS INTO THERAPEUTIC POSSIBILITIES IN NMOSD

The first‐ and second‐line therapies as well as recent developed new biological agents in NMOSD patients with AQP4‐IgG seronegative showed no clear therapeutic effect. Also some refractory patients with AQP4‐IgG seropositive did not respond to these treatments well. Although these new biological agents, such as satralizumab and inebilizumab, have obtained the positive results in the phase III, they still have some defects and deficiencies, such as small sample size and lack of long‐term data. Thus, it is necessary to further confirm the effectiveness and safety of the biological agents in treatment NMOSD through conducting more clinic trails in the near future. At the same time, it is a strong need to develop new target‐specific molecules related to the pathogenicity of NMOSD and it also requires development of diverging therapeutic demands for AQP4‐IgG‐seronegative patients.

### 3.1 Targeting Th17 cells and blocking IL23/IL17/Th17 pathway

Previous studies have found that pathogenic AQP4‐IgG can participate in the pathogenesis of NMOSD by activating complement, inducing inflammation, and destroying the BBB. However, the latest study found that some NMOSD patients do not have AQP4‐IgG, which suggests that there may be other pathogenic factors contributed to the occurrence of the disease. Among these pathogenic factors, T‐helper 17 (Th17) cells may be a crucial factor that cause the pathology in NMOSD. Based on the recent findings, it has been evidenced that elevated IL‐6 and IL‐17 are associated with severe disability of NMOSD and lower levels of IL‐6 and IL‐17 were found in patients after anti‐CD20 therapy.^82^ Additionally, higher proportion of Th17 cells and higher levels of IL‐17 as well as its related cytokines, IL‐6, IL‐21, and IL‐23 in both CSF and plasma were observed in NMOSD than in the control group in a meta‐analysis with 38 trials, indicating that Th17 cells and IL‐17 might play a pathogenic role in development of NMOSD and related to disease severity.^83^, ^84^ However, it is still unclear whether Th17 cells are the pathogenic cells in NMOSD without AQP4‐IgG, which requires further study. Th17 cells can produce the proinflammatory cytokines IL‐17 and IL‐23, and both of them can promote the differentiation of Th17 cells to drive autoimmune response and inflammation in several disorders, including NMOSD. IL23/IL17/Th17 pathway may be a central hub to NMOSD development and a key therapeutic target,^85^, ^86^ especially IL23/IL17/Th17 pathway, which may be involved in the pathogenesis of NMOSD without AQP4‐IgG. A better understanding of the roles of Th17 cells, IL‐17, and IL‐23 in NMOSD can provide new knowledge and help explore the promising and novel treatment strategy. Therefore, Th17 cells and IL‐17 may be new targets for therapy in NMOSD, particularly in NMOSD patients with AQP4‐IgG seronegative.^85^, ^86^ Furthermore, an increased Th2‐related cytokines IL‐25, IL‐31, and IL‐33 were observed in NMOSD. There was an association between serum level of IL‐33 in acute phase and past attacks, while lower serum level of IL‐35 was related to disease severity of NMOSD, which calls for conducting further studies to find more new therapeutic targets in NMOSD.^87^, ^88^

### 3.2 Stem cell therapy

The pluripotent stem cells derived from different tissues are a promising therapeutic approach to restore the damaged CNS functions since the stem cells can enhance self‐renewal or self‐replication ability. Stem cell therapy was successful to improve motor functions in several animal models of Parkinson's disease^89^ and spinal cord injury.^90^, ^91^ Stem cell therapy has beneficial effects on recovery from CNS injury, but the mechanisms of action remain not entirely clear.

Autologous hematopoietic stem cell transplantation (AHSCT) has been applied in treatment of MS, which is considered as a reconstitution form of immunotherapy, or a myeloablative and lymphoablative form of immunotherapy. In a recent study, three NMOSD patients received AHSCT with cyclophosphamide, rabbit antithymocyte globulin, and RTX and followed up for ≥5 years. The outcome showed that AHSCT seemed safe and effective with two patients showing improvement in disease activity and disability.^92^

In a prospective cohort study, 11 NMOSD patients with AQP4 IgG positive, 1 NMOSD without AQP4 IgG, and 1 NMOSD AQP4 IgG positive after cyclophosphamide treatment were enrolled. Unselected peripheral blood stem cells were transplanted to 13 patients and median follow‐up was nearly 5 years. The outcome showed that 80% patients were relapse free without any immunosuppression for more than 5 years post‐transplant with hematopoietic stem cell transplantation.^93^

One study also reported that a patient with NMOSD who underwent autologous peripheral hematopoietic stem cell transplantation (APHSCT) was followed up for 12 months with reduce the frequency of attacks and improvement in disability.^94^ Strikingly, two NMOSD patients with severe forms received APHSCT were observed AQP4‐IgG disappearance, sustained clinical remission, and radiological improvement as well as rebuilt of naive immune system.^92^ The curative and adverse effects of AHSCT on NMOSD were evaluated in 27 studies from the following databases (PubMed, Web of Science, Medline, EMBASE, Cochrane, and Google Scholar) and in a meta‐analysis with 31 NMOSD patients before December 2019.^95^ The results showed that AHSCT treatment resulted in long‐term effect on NMOSD patients with a high safety.^95^ Fifteen NMOSD patients received a single intravenous infusion of autologous bone marrow‐derived mesenchymal stem cells (MSCs) for 12 weeks for evaluation of the safety and efficacy of MSCs in NMOSD. The results demonstrated that MSC treatment for NMOSD patients was safe and effective in diminishing the relapse frequency and neurological disability for 2 years. The conclusion was that MSC treatment benefits NMOSD patients.^96^ Strikingly, the treatment with autologous stem cell transplantations in late‐stage refractory NMOSD patients led to seroconversion from AQP4‐IgG seropositivity to negativity without relapses.^97^, ^98^

Recently, Ceglie et al. summarized the therapeutic effects of hematopoietic stem cell transplantation (HSCT), including both autologous and allogeneic HSCT on NMOSD. They proposed that HSCT can induce tolerance to autoantigens and need to further explore graft versus autoimmunity effects, which will warrant to extend clinical trials to investigate this promising therapeutic option.^99^

### 3.3 Individualized therapy

Individualized management and therapy in NMOSD patients are essential and should be considered based on the above comprehensive studies and descriptions. Previous broad immunosuppression in NMOSD and now shifting to tailored treatments should to be promising efficient.^7^, ^70^ First, individualized therapy must be expanded to choose more treatments, including the new developed biologicals, since current effective treatments in acute attack of NMOSD resulted in complete recovery about 30% of attacks only.^100^, ^101^ We suggest that it should not be restricted to only use the first‐line or second‐line traditional drugs in NMOSD, we should based on the patient's response to treatment, including the efficacy, safety and tolerability of drugs, other drugs with different curative mechanisms might be considered to apply first.

In NMOSD patients, in whom AQP4 IgG is not a dominant pathogenic autoantigen, the blockers of IL23/IL17/Th17 pathway may be conducted in clinical trials after receiving a permission to explore the role of the blockers in the pathogenesis of NMOSD without AQP4‐IgG and understanding their therapeutic efficacy, which is a bit further away on the horizon probably. At the same time, it is necessary to detect anti‐MOG antibody in NMOSD patients without AQP4‐IgG, so that the NMOSD patients with MOG‐IgG can be treated accordingly.

Individualized therapy in NMOSD patients might also use combination therapy with PP and other therapies to maximize the therapeutic effect of their respective drugs, while minimizing side effects and adverse events. Other potential strategies, such as inducing immune tolerance via oral or nasal way, modulating T‐ and B‐cell functions, as well as inventing DNA vaccination, for individualization have been investigating.^65^, ^102^, ^103^, ^104^ Finally, a question needs to be answered in the near future, that is, when and whether long‐term therapy in NMOSD should be ceased in lack of disease activity?

One issue worthy of attention is that we should monitor the titers of AQP4‐IgG in order to observe the efficacy of the applied therapies and also further investigate the nexus between the levels of AQP4‐IgG and clinical severity and remission. In addition, there may be other molecules and factors involved in the pathogenesis of NMOSD, such as B‐lymphoid tyrosine kinase (BLK), and the polymorphism and mRNA gene expression of BLK may have impact on the sensitivity of NMOSD, therefore, it also needs to be further studied in the future.^105^

## 4 CONCLUSION

The latest advances in immunology, pathology, pharmacology and other disciplines have enabled us to have a deeper understanding of NMOSD, and also opened a new era of NMOSD treatment. On the basis of standard treatment, the amazing novel therapeutic biologicals with diverse curative targets and mechanisms have started to be applied in order to treat NMOSD patients and finally emerge as effective and promising therapeutic interventions. However, the efficacy of the novel therapeutic biologicals is still limited due to lack of large clinical data to confirm. Therefore, it is necessary to continue conducting clinical trials and developing new effective drugs for treatment of NMOSD in the near future.

## CONFLICTS OF INTEREST

None.

## AUTHORS’ CONTRIBUTIONS

MS, FC, and JZ prepared the manuscript; TJ and JZ provided views and revised the manuscript; JZ designed the framework of manuscript, prepared and finalized the manuscript. All authors agreed to approve the final manuscript.

## CONSENT FOR PUBLICATION

The manuscript has been approved by all the authors who are responsible for its content. The manuscript, including the figure, is original, and has not been submitted or published in any journal. All authors agree with the terms of the BioMed Central Copyright and License Agreement.

## Data Availability

Data sharing is not applicable to this article as no new data were created or analyzed in this study.

## References

[1] Oh J , Levy M . Neuromyelitis optica: an antibody‐mediated disorder of the central nervous system. Neurol Res Int. 2012;2012:460825. 22363840 10.1155/2012/460825 PMC3272864

[2] Wingerchuk DM , Banwell B , Bennett JL , et al. International consensus diagnostic criteria for neuromyelitis optica spectrum disorders. Neurology. 2015;85(2):177‐189. 26092914 10.1212/WNL.0000000000001729 PMC4515040

[3] Weinshenker BG , Wingerchuk DM . Neuromyelitis spectrum disorders. Mayo Clin Proc. 2017;92(4):663‐679. 28385199 10.1016/j.mayocp.2016.12.014

[4] Hor JY , Asgari N , Nakashima I , et al. Epidemiology of neuromyelitis optica spectrum disorder and its prevalence and incidence worldwide. Front Neurol. 2020;11:501. 32670177 10.3389/fneur.2020.00501 PMC7332882

[5] Cree BAC , Bennett JL , Kim HJ , et al. Inebilizumab for the treatment of neuromyelitis optica spectrum disorder (N‐MOmentum): a double‐blind, randomised placebo‐controlled phase 2/3 trial. Lancet (London, England). 2019;394(10206):1352‐1363. 10.1016/S0140-6736(19)31817-3 31495497

[6] Asseyer S , Schmidt F , Chien C , et al. Pain in AQP4‐IgG‐positive and MOG‐IgG‐positive neuromyelitis optica spectrum disorders. Multiple Sclerosis J ‐ Experimental, Transl Clin. 2018;4(3):2055217318796684. 10.1177/2055217318796684 PMC6117869 30186620

[7] Hyun JW , Jang H , Yu J , et al. Comparison of neuropathic pain in neuromyelitis optica spectrum disorder and multiple sclerosis. J Clin Neurol (Seoul, Korea). 2020;16(1):124‐130. 10.3988/jcn.2020.16.1.124 PMC6974826 31942768

[8] Pandit L . Neuromyelitis optica spectrum disorders: an update. Ann Indian Acad Neurol. 2015;18(Suppl 1):S11‐S15. 26538842 10.4103/0972-2327.164816 PMC4604691

[9] Wu Y , Zhong L , Geng J . Neuromyelitis optica spectrum disorder: Pathogenesis, treatment, and experimental models. Mult Scler Relat Disord. 2019;27:412‐418. 30530071 10.1016/j.msard.2018.12.002

[10] Etemadifar M , Nasr Z , Khalili B , Taherioun M , Vosoughi R . Epidemiology of neuromyelitis optica in the world: a systematic review and meta‐analysis. Multiple Sclerosis Int. 2015;2015:174720. 10.1155/2015/174720 PMC4417948 25973275

[11] Bukhari W , Prain KM , Waters P , et al. Incidence and prevalence of NMOSD in Australia and New Zealand. J Neurol Neurosurg Psychiatry. 2017;88(8):632‐638. 28550069 10.1136/jnnp-2016-314839

[12] Lennon VA , Wingerchuk DM , Kryzer TJ , et al. A serum autoantibody marker of neuromyelitis optica: distinction from multiple sclerosis. Lancet (London, England). 2004;364(9451):2106‐2112. 10.1016/S0140-6736(04)17551-X 15589308

[13] Lennon VA , Kryzer TJ , Pittock SJ , Verkman AS , Hinson SR . IgG marker of optic‐spinal multiple sclerosis binds to the aquaporin‐4 water channel. J Exp Med. 2005;202(4):473‐477. 16087714 10.1084/jem.20050304 PMC2212860

[14] Papadopoulos MC , Verkman AS . Aquaporin 4 and neuromyelitis optica. Lancet Neurol. 2012;11(6):535‐544. 22608667 10.1016/S1474-4422(12)70133-3 PMC3678971

[15] Misu T , Fujihara K , Kakita A , et al. Loss of aquaporin 4 in lesions of neuromyelitis optica: distinction from multiple sclerosis. Brain. 2007;130(Pt 5):1224‐1234. 17405762 10.1093/brain/awm047

[16] Roemer SF , Parisi JE , Lennon VA , et al. Pattern‐specific loss of aquaporin‐4 immunoreactivity distinguishes neuromyelitis optica from multiple sclerosis. Brain. 2007;130(Pt 5):1194‐1205. 17282996 10.1093/brain/awl371

[17] Jarius S , Wildemann B , Paul F . Neuromyelitis optica: clinical features, immunopathogenesis and treatment. Clin Exp Immunol. 2014;176(2):149‐164. 24666204 10.1111/cei.12271 PMC3992027

[18] Ferrán C , Pedemonte V , Turcatti E , González G . Neuromyelitis optica. Medicina. 2019;79(Suppl 3):60‐65. 31603846

[19] Chen JJ , Bhatti MT . Clinical phenotype, radiological features, and treatment of myelin oligodendrocyte glycoprotein‐immunoglobulin G (MOG‐IgG) optic neuritis. Curr Opin Neurol. 2020;33(1):47‐54. 31743235 10.1097/WCO.0000000000000766

[20] Carnero Contentti E , Lopez PA , Pettinicchi JP , et al. What percentage of AQP4‐ab‐negative NMOSD patients are MOG‐ab positive? A study from the Argentinean multiple sclerosis registry (RelevarEM). Mult Scler Relat Disord. 2021;49:102742. 33454601 10.1016/j.msard.2021.102742

[21] Yan Y , Li Y , Fu Y , et al. Autoantibody to MOG suggests two distinct clinical subtypes of NMOSD. Sci China Life Sci. 2016;59(12):1270‐1281. 26920678 10.1007/s11427-015-4997-y PMC5101174

[22] Gospe SM 3rd , Chen JJ , Bhatti MT . Neuromyelitis optica spectrum disorder and myelin oligodendrocyte glycoprotein associated disorder‐optic neuritis: a comprehensive review of diagnosis and treatment. Eye (London, England). 2021;35(3):753‐768. 10.1038/s41433-020-01334-8 PMC8026985 33323985

[23] Liu C , Shi M , Zhu M , Chu F , Jin T , Zhu J . Comparisons of clinical phenotype, radiological and laboratory features, and therapy of neuromyelitis optica spectrum disorder by regions: update and challenges. Autoimmun Rev. 2021;21(1):102921. 34384938 10.1016/j.autrev.2021.102921

[24] Tradtrantip L , Jin BJ , Yao X , Anderson MO , Verkman AS . Aquaporin‐targeted therapeutics: state‐of‐the‐field. Adv Exp Med Biol. 2017;969:239‐250. 28258578 10.1007/978-94-024-1057-0_16 PMC5628614

[25] Akaishi T , Nakashima I , Sato DK , Takahashi T , Fujihara K . Neuromyelitis optica spectrum disorders. Neuroimaging Clin N Am. 2017;27(2):251‐265. 28391784 10.1016/j.nic.2016.12.010

[26] Rosenthal JF , Hoffman BM , Tyor WR . CNS inflammatory demyelinating disorders: MS, NMOSD and MOG antibody associated disease. J Investig Med. 2020;68(2):321‐330. 10.1136/jim-2019-001126 31582425

[27] Trebst C , Jarius S , Berthele A , et al. Update on the diagnosis and treatment of neuromyelitis optica: recommendations of the Neuromyelitis Optica Study Group (NEMOS). J Neurol. 2014;261(1):1‐16. 10.1007/s00415-013-7169-7 PMC3895189 24272588

[28] Poupart J , Giovannelli J , Deschamps R , et al. Evaluation of efficacy and tolerability of first‐line therapies in NMOSD. Neurology. 2020;94(15):e1645‐e1656. 32170036 10.1212/WNL.0000000000009245

[29] Chen H , Qiu W , Zhang Q , et al. Comparisons of the efficacy and tolerability of mycophenolate mofetil and azathioprine as treatments for neuromyelitis optica and neuromyelitis optica spectrum disorder. Eur J Neurol. 2017;24(1):219‐226. 27783452 10.1111/ene.13186

[30] Pittock SJ , Berthele A , Fujihara K , et al. Eculizumab in Aquaporin‐4‐positive neuromyelitis optica spectrum disorder. N Engl J Med. 2019;381(7):614‐625. 31050279 10.1056/NEJMoa1900866

[31] Zhang C , Zhang M , Qiu W , et al. Safety and efficacy of tocilizumab versus azathioprine in highly relapsing neuromyelitis optica spectrum disorder (TANGO): an open‐label, multicentre, randomised, phase 2 trial. Lancet Neurol. 2020;19(5):391‐401. 32333897 10.1016/S1474-4422(20)30070-3 PMC7935423

[32] Traboulsee A , Greenberg BM , Bennett JL , et al. Safety and efficacy of satralizumab monotherapy in neuromyelitis optica spectrum disorder: a randomised, double‐blind, multicentre, placebo‐controlled phase 3 trial. Lancet Neurol. 2020;19(5):402‐412. 32333898 10.1016/S1474-4422(20)30078-8 PMC7935419

[33] Levy M , Fujihara K , Palace J . New therapies for neuromyelitis optica spectrum disorder. Lancet Neurol. 2021;20(1):60‐67. 33186537 10.1016/S1474-4422(20)30392-6

[34] Wang X , Jiao W , Lin M , et al. Resolution of inflammation in neuromyelitis optica spectrum disorders. Mult Scler Relat Disord. 2019;27:34‐41. 30300851 10.1016/j.msard.2018.09.040

[35] Wang Y , Zhu M , Liu C , et al. Blood brain barrier permeability could be a biomarker to predict severity of neuromyelitis optica spectrum disorders: a retrospective analysis. Front Neurol. 2018;9:648. 30131763 10.3389/fneur.2018.00648 PMC6090143

[36] Liang H , Gao W , Liu X , et al. The GTF2I rs117026326 polymorphism is associated with neuromyelitis optica spectrum disorder but not with multiple sclerosis in a Northern Han Chinese population. J Neuroimmunol. 2019;337:577045. 31520790 10.1016/j.jneuroim.2019.577045

[37] Yang MG , Tian S , Zhang Q , et al. Elevated serum interleukin‐39 levels in patients with neuromyelitis optica spectrum disorders correlated with disease severity. Mult Scler Relat Disord. 2020;46:102430. 32853892 10.1016/j.msard.2020.102430

[38] Song W , Qu Y , Huang X . Plasma exchange: an effective add‐on treatment of optic neuritis in neuromyelitis optica spectrum disorders. Int Ophthalmol. 2019;39(11):2477‐2483. 30825049 10.1007/s10792-019-01090-z

[39] Lin CW , Lin IH , Chen TC , Jou JR , Woung LC . Clinical course and treatment response of neuromyelitis optica spectrum disease: An 8‐year experience. Asia‐Pacific J Ophthalmol (Philadelphia, Pa). 2019;8(3):206‐210. 10.22608/APO.2018247 30421588

[40] Batra A , Periyavan S . Role of low plasma volume treatment on clinical efficacy of plasmapheresis in neuromyelitis optica. Asian J Transfusion Sci. 2017;11(2):102‐107. 10.4103/ajts.AJTS_111_16 PMC5613414 28970675

[41] Ong ZM , Schee JP , Viswanathan S . Therapeutic plasma exchange in neuromyelitis optica spectrum disorders and related disorders in resource‐limited settings: outcomes in a multiethnic single‐center population. Ther Apher Dial. 2020;24(3):312‐323. 31654607 10.1111/1744-9987.13446

[42] Oshiro A , Nakamura S , Tamashiro K , Fujihara K . Anti‐MOG + neuromyelitis optica spectrum disorders treated with plasmapheresis. No to Hattatsu = Brain Development. 2016;48(3):199‐203. 27349083

[43] Aungsumart S , Apiwattanakul M . Clinical outcomes and predictive factors related to good outcomes in plasma exchange in severe attack of NMOSD and long extensive transverse myelitis: case series and review of the literature. Mult Scler Relat Disord. 2017;13:93‐97. 28427710 10.1016/j.msard.2017.02.015

[44] Loken MR , Shah VO , Dattilio KL , Civin CI . Flow cytometric analysis of human bone marrow. II. Normal B lymphocyte development. Blood. 1987;70(5):1316‐1324. 3117132

[45] Cabre P , Mejdoubi M , Jeannin S , et al. Treatment of neuromyelitis optica with rituximab: a 2‐year prospective multicenter study. J Neurol. 2018;265(4):917‐925. 29455361 10.1007/s00415-018-8771-5

[46] Chamberlain N , Massad C , Oe T , Cantaert T , Herold KC , Meffre E . Rituximab does not reset defective early B cell tolerance checkpoints. J Clin Invest. 2016;126(1):282‐287. 26642366 10.1172/JCI83840 PMC4701568

[47] Tahara M , Oeda T , Okada K , et al. Safety and efficacy of rituximab in neuromyelitis optica spectrum disorders (RIN‐1 study): a multicentre, randomised, double‐blind, placebo‐controlled trial. Lancet Neurol. 2020;19(4):298‐306. 32199095 10.1016/S1474-4422(20)30066-1

[48] Correa‐Díaz EP , Torres‐Herrán GE , Miño Zambrano JE , Paredes‐Gonzalez V , Caiza‐Zambrano FJ . Impact of Rituximab on relapse rate and disability in an Ecuadorian cohort of patients with neuromyelitis optica spectrum disorders. Mult Scler Relat Disord. 2021;48:102683. 33338945 10.1016/j.msard.2020.102683

[49] Kim SH , Huh SY , Jang H , et al. Outcome of pregnancies after onset of the neuromyelitis optica spectrum disorder. Eur J Neurol. 2020;27(8):1546‐1555. 32320109 10.1111/ene.14274

[50] Kim SH , Jeong IH , Hyun JW , et al. Treatment outcomes with rituximab in 100 patients with neuromyelitis optica: influence of FCGR3A polymorphisms on the therapeutic response to rituximab. JAMA Neurol. 2015;72(9):989‐995. 26167726 10.1001/jamaneurol.2015.1276

[51] Kunchok A , Malpas C , Nytrova P , et al. Clinical and therapeutic predictors of disease outcomes in AQP4‐IgG+ neuromyelitis optica spectrum disorder. Mult Scler Relat Disord. 2020;38:101868. 31877445 10.1016/j.msard.2019.101868

[52] Kleiter I , Gold R . Present and future therapies in neuromyelitis optica spectrum disorders. Neurotherapeutics. 2016;13(1):70‐83. 26597098 10.1007/s13311-015-0400-8 PMC4720663

[53] Shi Z , Du Q , Chen H , et al. Effects of immunotherapies and prognostic predictors in neuromyelitis optica spectrum disorder: a prospective cohort study. J Neurol. 2020;267(4):913‐924. 31776721 10.1007/s00415-019-09649-7

[54] Yang Y , Wang CJ , Wang BJ , Zeng ZL , Guo SG . Comparison of efficacy and tolerability of azathioprine, mycophenolate mofetil, and lower dosages of rituximab among patients with neuromyelitis optica spectrum disorder. J Neurol Sci. 2018;385:192‐197. 29406904 10.1016/j.jns.2017.12.034

[55] Xu Y , Wang Q , Ren HT , et al. Comparison of efficacy and tolerability of azathioprine, mycophenolate mofetil, and cyclophosphamide among patients with neuromyelitis optica spectrum disorder: A prospective cohort study. J Neurol Sci. 2016;370:224‐228. 27772764 10.1016/j.jns.2016.09.035

[56] Ramanathan RS , Malhotra K , Scott T . Treatment of neuromyelitis optica/neuromyelitis optica spectrum disorders with methotrexate. BMC Neurol. 2014;14:51. 24628894 10.1186/1471-2377-14-51 PMC3985587

[57] Kageyama T , Komori M , Miyamoto K , et al. Combination of cyclosporine A with corticosteroids is effective for the treatment of neuromyelitis optica. J Neurol. 2013;260(2):627‐634. 23076828 10.1007/s00415-012-6692-2

[58] Yaguchi H , Sakushima K , Takahashi I , et al. Efficacy of intravenous cyclophosphamide therapy for neuromyelitis optica spectrum disorder. Int Med (Tokyo, Japan). 2013;52(9):969‐972. 10.2169/internalmedicine.52.7885 23648715

[59] Zhang Y , Fei Y , Niu J , et al. Retrospective study of clinical features of neuromyelitis optica spectrum disease with connective tissue disease. Zhonghua Yi Xue Za Zhi. 2014;94(39):3056‐3061. 25549677

[60] Huang W , Wang L , Zhang B , Zhou L , Zhang T , Quan C . Effectiveness and tolerability of immunosuppressants and monoclonal antibodies in preventive treatment of neuromyelitis optica spectrum disorders: A systematic review and network meta‐analysis. Mult Scler Relat Disord. 2019;35:246‐252. 31425902 10.1016/j.msard.2019.08.009

[61] Chihara N , Aranami T , Sato W , et al. Interleukin 6 signaling promotes anti‐aquaporin 4 autoantibody production from plasmablasts in neuromyelitis optica. Proc Natl Acad Sci USA. 2011;108(9):3701‐3706. 21321193 10.1073/pnas.1017385108 PMC3048150

[62] Franciotta D , Salvetti M , Lolli F , Serafini B , Aloisi F . B cells and multiple sclerosis. Lancet Neurol. 2008;7(9):852‐858. 18703007 10.1016/S1474-4422(08)70192-3

[63] Traub J , Häusser‐Kinzel S , Weber MS . Differential effects of MS therapeutics on B cells‐implications for their use and failure in AQP4‐positive NMOSD patients. Int J Mol Sci. 2020;21(14):5021. 10.3390/ijms21145021 PMC7404039 32708663

[64] Graf J , Mares J , Barnett M , et al. Targeting B cells to modify MS, NMOSD, and MOGAD: Part 2. Neurology(R) Neuroimmunol Neuroinflamm. 2021;8(1):e919. 10.1212/NXI.0000000000000919 PMC8063618 33411674

[65] Wallach AI , Tremblay M , Kister I . Advances in the treatment of neuromyelitis optica spectrum disorder. Neurol Clin. 2021;39(1):35‐49. 33223088 10.1016/j.ncl.2020.09.003

[66] DiLillo DJ , Hamaguchi Y , Ueda Y , et al. Maintenance of long‐lived plasma cells and serological memory despite mature and memory B cell depletion during CD20 immunotherapy in mice. J Immunol. 2008;180(1):361‐371. 18097037 10.4049/jimmunol.180.1.361

[67] Kümpfel T , Thiel S , Meinl I , et al. Anti‐CD20 therapies and pregnancy in neuroimmunologic disorders: a cohort study from Germany. Neurology(R) Neuroimmunol Neuroinflamm. 2021;8(1):e913. 10.1212/NXI.0000000000000913 PMC7757754 33334856

[68] Carreón Guarnizo E , Hernández Clares R , Castillo Triviño T , et al. Experience with tocilizumab in patients with neuromyelitis optica spectrum disorders. Neurologia (Barcelona, Spain) 2019;S0213‐4853(19):30033‐7. 10.1016/j.nrleng.2018.12.021 35465911

[69] Xie Q , Zheng T , Sun M , Sun J , Wang M . A meta‐analysis to determine the efficacy and safety of tocilizumab in neuromyelitis optica spectrum disorders. Mult Scler Relat Disord. 2020;45:102421. 32731203 10.1016/j.msard.2020.102421

[70] Duchow A , Bellmann‐Strobl J . Satralizumab in the treatment of neuromyelitis optica spectrum disorder. Neurodegenerative Dis Manage. 2021;11(1):49‐59. 10.2217/nmt-2020-0046 33167776

[71] Rigal J , Pugnet G , Ciron J , Lépine Z , Biotti D . Off‐label use of tocilizumab in neuromyelitis optica spectrum disorders and MOG‐antibody‐associated diseases: a case‐series. Mult Scler Relat Disord. 2020;46:102483. 32942119 10.1016/j.msard.2020.102483

[72] Araki M . Blockade of IL‐6 signaling in neuromyelitis optica. Neurochem Int. 2019;130:104315. 30342072 10.1016/j.neuint.2018.10.012

[73] Lotan I , Charlson RW , Ryerson LZ , Levy M , Kister I . Effectiveness of subcutaneous tocilizumab in neuromyelitis optica spectrum disorders. Mult Scler Relat Disord. 2019;39:101920. 31918241 10.1016/j.msard.2019.101920

[74] Yamamura T , Kleiter I , Fujihara K , et al. Trial of satralizumab in neuromyelitis optica spectrum disorder. N Engl J Med. 2019;381(22):2114‐2124. 31774956 10.1056/NEJMoa1901747

[75] Pittock SJ , Lennon VA , McKeon A , et al. Eculizumab in AQP4‐IgG‐positive relapsing neuromyelitis optica spectrum disorders: an open‐label pilot study. Lancet Neurol. 2013;12(6):554‐562. 23623397 10.1016/S1474-4422(13)70076-0

[76] Palace J , Wingerchuk DM , Fujihara K , et al. Benefits of eculizumab in AQP4+ neuromyelitis optica spectrum disorder: Subgroup analyses of the randomized controlled phase 3 PREVENT trial. Mult Scler Relat Disord. 2020;47:102641. 33310418 10.1016/j.msard.2020.102641

[77] Namatame C , Misu T , Takai Y , et al. CH50 as a putative biomarker of eculizumab treatment in neuromyelitis optica spectrum disorder. Heliyon. 2021;7(1):e05899. 33490671 10.1016/j.heliyon.2021.e05899 PMC7809378

[78] Paul F , Murphy O , Pardo S , Levy M . Investigational drugs in development to prevent neuromyelitis optica relapses. Expert Opin Investig Drugs. 2018;27(3):265‐271. 10.1080/13543784.2018.1443077 29465257

[79] Holmøy T , Høglund RA , Illes Z , Myhr KM , Torkildsen Ø . Recent progress in maintenance treatment of neuromyelitis optica spectrum disorder. J Neurol. 2020;268(12):4522‐4536. 33011853 10.1007/s00415-020-10235-5 PMC8563615

[80] Holroyd KB , Manzano GS , Levy M . Update on neuromyelitis optica spectrum disorder. Curr Opin Ophthalmol. 2020;31(6):462‐468. 33009077 10.1097/ICU.0000000000000703 PMC7771018

[81] Traub J , Husseini L , Weber MS . B cells and antibodies as targets of therapeutic intervention in neuromyelitis optica spectrum disorders. Pharmaceuticals (Basel, Switzerland). 2021;14(1):37. 10.3390/ph14010037 PMC7825598 33419217

[82] Agasing AM , Wu Q , Khatri B , et al. Transcriptomics and proteomics reveal a cooperation between interferon and T‐helper 17 cells in neuromyelitis optica. Nat Commun. 2020;11(1):2856. 32503977 10.1038/s41467-020-16625-7 PMC7275086

[83] Barros PO , Dias ASO , Kasahara TM , et al. Expansion of IL‐6(+) Th17‐like cells expressing TLRs correlates with microbial translocation and neurological disabilities in NMOSD patients. J Neuroimmunol. 2017;307:82‐90. 28495144 10.1016/j.jneuroim.2017.04.001

[84] Hou MM , Li YF , He LL , et al. Proportions of Th17 cells and Th17‐related cytokines in neuromyelitis optica spectrum disorders patients: A meta‐analysis. Int Immunopharmacol. 2019;75:105793. 31401379 10.1016/j.intimp.2019.105793

[85] Lin J , Li X , Xia J . Th17 cells in neuromyelitis optica spectrum disorder: a review. Int J Neurosci. 2016;126(12):1051‐1060. 10.3109/00207454.2016.1163550 26954363

[86] Klein da Costa B , de Souza B , Melo R , et al. Unraveling B lymphocytes in CNS inflammatory diseases: Distinct mechanisms and treatment targets. Neurology. 2020;95(16):733‐744. 32907966 10.1212/WNL.0000000000010789

[87] Zhang Y , Yao XY , Gao MC , et al. Th2 axis‐related cytokines in patients with neuromyelitis optica spectrum disorders. CNS Neurosci Ther. 2018;24(1):64‐69. 29110391 10.1111/cns.12774 PMC6490054

[88] Yang H , Han L , Zhou YJ , et al. Lower serum interleukin‐22 and interleukin‐35 levels are associated with disease status in neuromyelitis optica spectrum disorders. CNS Neurosci Ther. 2020;26(2):251‐259. 31342670 10.1111/cns.13198 PMC6978267

[89] Morizane A , Li JY , Brundin P . From bench to bed: the potential of stem cells for the treatment of Parkinson's disease. Cell Tissue Res. 2008;331(1):323‐336. 18034267 10.1007/s00441-007-0541-0

[90] Fujimoto Y , Abematsu M , Falk A , et al. Treatment of a mouse model of spinal cord injury by transplantation of human induced pluripotent stem cell‐derived long‐term self‐renewing neuroepithelial‐like stem cells. Stem Cells. 2012;30(6):1163‐1173. 22419556 10.1002/stem.1083

[91] Tetzlaff W , Okon EB , Karimi‐Abdolrezaee S , et al. A systematic review of cellular transplantation therapies for spinal cord injury. J Neurotrauma. 2010;28(8):1611‐1682. 20146557 10.1089/neu.2009.1177 PMC3143488

[92] Burton JM , Duggan P , Costello F , Metz L , Storek J . A pilot trial of autologous hematopoietic stem cell transplant in neuromyelitis optic spectrum disorder. Mult Scler Relat Disord. 2021;53:102990. 34082329 10.1016/j.msard.2021.102990

[93] Burt RK , Balabanov R , Han X , et al. Autologous nonmyeloablative hematopoietic stem cell transplantation for neuromyelitis optica. Neurology. 2019;93(18):e1732‐e1741. 31578302 10.1212/WNL.0000000000008394 PMC6946475

[94] Peng F , Qiu W , Li J , et al. A preliminary result of treatment of neuromyelitis optica with autologous peripheral hematopoietic stem cell transplantation. Neurologist. 2010;16(6):375‐378. 21150387 10.1097/NRL.0b013e3181b126e3

[95] Zhang P , Liu B . Effect of autologous hematopoietic stem cell transplantation on multiple sclerosis and neuromyelitis optica spectrum disorder: a PRISMA‐compliant meta‐analysis. Bone Marrow Transplant. 2020;55(10):1928‐1934. 32020080 10.1038/s41409-020-0810-z

[96] Fu Y , Yan Y , Qi Y , et al. Impact of autologous mesenchymal stem cell infusion on neuromyelitis optica spectrum Disorder: a pilot, 2‐year observational study. CNS Neurosci Ther. 2016;22(8):677‐685. 27219819 10.1111/cns.12559 PMC6492909

[97] Oertel FC , Havla J , Roca‐Fernández A , et al. Retinal ganglion cell loss in neuromyelitis optica: a longitudinal study. J Neurol Neurosurg Psychiatry. 2018;89(12):1259‐1265. 29921610 10.1136/jnnp-2018-318382

[98] Oertel FC , Outteryck O , Knier B , et al. Optical coherence tomography in myelin‐oligodendrocyte‐glycoprotein antibody‐seropositive patients: a longitudinal study. J Neuroinflamm. 2019;16(1):154. 10.1186/s12974-019-1521-5 PMC6657100 31345223

[99] Ceglie G , Papetti L , Valeriani M , Merli P . Hematopoietic stem cell transplantation in neuromyelitis optica‐spectrum disorders (NMO‐SD): state‐of‐the‐art and future perspectives. Int J Mol Sci. 2020;21(15):5304. 10.3390/ijms21155304 PMC7432050 32722601

[100] Elsone L , Kitley J , Luppe S , et al. Long‐term efficacy, tolerability and retention rate of azathioprine in 103 aquaporin‐4 antibody‐positive neuromyelitis optica spectrum disorder patients: a multicentre retrospective observational study from the UK. Multiple Sclerosis. 2014;20(11):1533‐1540. 24647557 10.1177/1352458514525870

[101] Yamamura T , Nakashima I . Foveal thinning in neuromyelitis optica: A sign of retinal astrocytopathy? Neurology(R) Neuroimmunol Neuroinflamm. 2017;4(3):e347. 10.1212/NXI.0000000000000347 PMC5395067 28439528

[102] Ventura RE , Kister I , Chung S , Babb JS , Shepherd TM . Cervical spinal cord atrophy in NMOSD without a history of myelitis or MRI‐visible lesions. Neurology(R) Neuroimmunol Neuroinflamm. 2016;3(3):e224. 10.1212/NXI.0000000000000224 PMC4841642 27144215

[103] Oertel FC , Kuchling J , Zimmermann H , et al. Microstructural visual system changes in AQP4‐antibody‐seropositive NMOSD. Neurology(R) Neuroimmunol Neuroinflamm. 2017;4(3):e334. 10.1212/NXI.0000000000000334 PMC5322864 28255575

[104] Streicher K , Sridhar S , Kuziora M , et al. Baseline plasma cell gene signature predicts improvement in systemic sclerosis skin scores following treatment with inebilizumab (MEDI‐551) and correlates with disease activity in systemic lupus erythematosus and chronic obstructive pulmonary disease. Arthritis Rheumatol. 2018;70(12):2087‐2095. 29956883 10.1002/art.40656

[105] Yin BW , Li B , Mehmood A , et al. BLK polymorphisms and expression level in neuromyelitis optica spectrum disorder. CNS Neurosci Ther. 2021;27(12):1549‐1560. 34637583 10.1111/cns.13738 PMC8611770

[106] Schett G . Physiological effects of modulating the interleukin‐6 axis. Rheumatology (Oxford). 2018;57(suppl_2):ii43‐ii50. 29982781 10.1093/rheumatology/kex513

[107] Barros PO , Cassano T , Hygino J , et al. Prediction of disease severity in neuromyelitis optica by the levels of interleukin (IL)‐6 produced during remission phase. Clin Exp Immunol. 2016;183(3):480‐489. 26472479 10.1111/cei.12733 PMC4750605

